# Proteomic analysis of *Plasmodium falciparum* parasites from patients with cerebral and uncomplicated malaria

**DOI:** 10.1038/srep26773

**Published:** 2016-06-01

**Authors:** Gwladys I. Bertin, Audrey Sabbagh, Nicolas Argy, Virginie Salnot, Sem Ezinmegnon, Gino Agbota, Yélé Ladipo, Jules M. Alao, Gratien Sagbo, François Guillonneau, Philippe Deloron

**Affiliations:** 1 Institut de Recherche pour le Développement (IRD), UMR216 - MERIT, Paris, France; 2 COMUE Sorbonne Paris Cité, Faculté de Pharmacie de Paris, Paris Descartes University, Paris 75006, France; 3 Parasitology laboratory, Bichat-Claude Bernard hospital, Paris 75018, France; 4 French national reference center of malaria laboratory, Bichat-Claude Bernard hospital, Paris 75018, France; 5 3P5 Proteomics facility, Université Paris Descartes, Paris, France; 6 Centre d'Étude et de Recherche sur le Paludisme Associé à la Grossesse et l’Enfance (CERPAGE), Cotonou, Benin; 7 Paediatric Department, Mother and child hospital (HOMEL), Cotonou, Benin; 8 Paediatric Department, Centre National Hospitalo-Universitaire (CNHU), Cotonou, Benin

## Abstract

*Plasmodium falciparum* is responsible of severe malaria, including cerebral malaria (CM). During its intra-erythrocytic maturation, parasite-derived proteins are expressed, exported and presented at the infected erythrocyte membrane. To identify new CM-specific parasite membrane proteins, we conducted a mass spectrometry-based proteomic study and compared the protein expression profiles between 9 CM and 10 uncomplicated malaria (UM) samples. Among the 1097 *Plasmodium* proteins identified, we focused on the 499 membrane-associated and hypothetical proteins for comparative analysis. Filter-based feature selection methods combined with supervised data analysis identified a subset of 29 proteins distinguishing CM and UM samples with high classification accuracy. A hierarchical clustering analysis of these 29 proteins based on the similarity of their expression profiles revealed two clusters of 15 and 14 proteins, respectively under- and over-expressed in CM. Among the over-expressed proteins, the MESA protein is expressed at the erythrocyte membrane, involved in proteins trafficking and in the export of variant surface antigens (VSAs), but without antigenic function. Antigen 332 protein is exported at the erythrocyte, also involved in protein trafficking and in VSAs export, and exposed to the immune system. Our proteomics data demonstrate an association of selected proteins in the pathophysiology of CM.

In 2014, the World Health Organization reported an estimated 198 million cases of malaria worldwide, and 584,000 subsequent deaths from the disease, mostly among children below five years of age. *Plasmodium falciparum* is responsible of most of the severe forms, such as cerebral malaria (CM) or pregnancy-associated malaria (PAM). During the intra-erythrocytic proliferation of *P. falciparum*, parasite-derived proteins are successively expressed, exported, and presented at the surface of the human erythrocyte membrane, leading to physical and morphological alterations of the infected cell^1^,^2^. The shape and deformability of the infected erythrocyte (iE) is modified, and electron dense protrusions known as knobs appear at the iE surface^2^. The knobs structure involves complex interactions between parasite proteins and the iE membrane cytoskeleton^3^,^4^,^5^. The Knob-Associated Histidine Rich Protein (KAHRP) is the major plasmodial protein present in knobs^5^, and is involved in the export of variant surface antigens^6^,^7^. KAHRP also interacts with various cytoskeletal components of the erythrocyte membrane, including spectrin and actin^7^,^9^, leading to alterations of the iE membrane^1^,^8^. Another major knob protein is the Mature parasite-infected Erythrocyte Surface Antigen (MESA) or *Pf*EMP-2, involved in destabilizing the erythrocyte membrane skeleton through direct binding between *Pf*EMP-2, spectrin and actin^9^,^10^. These knob proteins are exported at iE membrane by Maurer’s clefts located in the sub-membrane region of iEs^11^.

Few studies have investigated the transcriptome of CM samples, and most focused on VSAs genes^12^,^13^. A single study has compared the transcriptomic and proteomic data from CM and PAM samples, showing that four genes were preferentially expressed in CM parasites: the genes encoding for the glutamic acid-rich protein, *Pf*EMP-2, the glycophorin-binding protein 2, and a putative protein^14^. However, the comparison between CM and PAM may not be the most appropriate, given the extent of differences as regards to parasite phenotype and immunological status of individuals.

The dogma that “one gene gives one protein” is often biased by translational and post- translational modifications. It is thus important to validate transcriptomic approaches at the proteomic level. In the case of CM, few studies have investigated the transcriptomic or proteomic pattern of field isolates^14^,^15^ as opposed to PAM where most studies involved such field isolates^16^,^17^,^18^. Using a proteomic approach, we compared the expression profiles of parasite proteins located in the infected-erythrocyte membrane or proteins of unknown function that are specific of *P. falciparum* isolates associated with two distinct clinical presentations of malaria, CM and uncomplicated malaria (UM).

## Results

### Descriptive data

**Figure 1:**
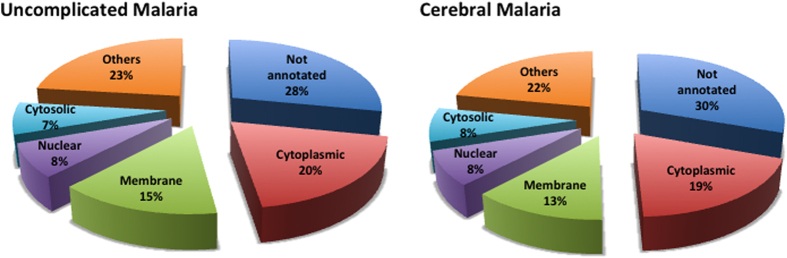
Proportion of proteins identified according to their cellular localization, by clinical group. Each part corresponds to one category of proteins: proteins identified in uncomplicated malaria samples and in cerebral malaria samples.

Ten and nine parasite samples from UM and CM cases respectively were analyzed by LC-MS/MS, and proteins were identified using two database search algorithms. Nine percent of the proteins were identified by a single algorithm (0 and 276 proteins were unique to Mascot and Sequest, respectively), and both algorithms identified 1527 proteins by combining erythrocyte proteins (n = 430) and parasite proteins (n = 1097), ensuring the reliability of protein identification. The numbers of proteins identified in samples from the two clinical groups were roughly similar (818 for CM *vs.* 965 for UM). The number of *Plasmodium* proteins (612 *vs.* 672) and their cellular localization were also comparable between CM and UM samples. Membrane proteins represented ~14% of all proteins in both groups; about 30% of proteins were not annotated (Fig. 1).

We identified the KAHRP and *Pf*EMP-3 proteins in all samples with a number of peptides of (12–24) and (4–22) in UM, and (9–30) and (3–21) in CM, respectively, demonstrating the ability to identify membrane proteins. Parasite proteins expressed at the iE membrane were identified in all samples.

### Identification of proteins differentially expressed between the two parasite populations

Differences in protein expression profiles between CM and UM samples were evaluated using a semi-quantitative approach. Mass spectrometric data were quantified by a validated peptide count analysis that computed emPAI values for all proteins, providing an estimation of the protein abundance^19^. Among the 1097 Plasmodium proteins identified in at least one sample with at least two peptides, we focused on the 499 proteins (45%) identified as either membrane-associated (160 membrane proteins) or hypothetical (339 putative proteins). The 598 proteins excluded from the analysis were mainly “housekeeping” proteins (ribosomal proteins) cytoplasmic and mitochondrial proteins.

The expression data were submitted to feature selection and supervised classification analysis to identify the subset of proteins that best discriminate between clinical forms of malaria.

We used a combination of three filter-based methods to select the proteins with differential expression profiles between CM and UM samples. From the original set of 499 proteins, Fisher filtering, Runs filtering and ReliefF selected a subset of 81, 6 and 37 proteins, respectively (Figure S1). We compiled these results to generate a new list composed of the proteins selected by at least two filter techniques. This led to a fourth list of 29 proteins (described in Figure S1). The performance of the different feature subsets to distinguish samples according to the clinical status of the donors was evaluated with supervised classification methods. The expression vectors (i.e., the pattern of protein expression in all samples) of the discriminatory proteins were used to train a classifier, and the ability of each protein subset to assign the correct class label (CM or UM) to any new sample was evaluated through a LOOCV procedure. Since different types of classifiers can respond differently to the same input data, four different classification tools were used, including *k*-nearest neighbors (*k*NN), multilayer perceptron neural network (MPNN), radial basis function neural network (RBFNN), and support vector machine (SVM). We estimated the prediction accuracy of the list of 29 proteins together with the three protein lists produced by the individual filters, and compared the results with those obtained with the initial set of 499 proteins (Table 1). Selecting a subset of features among all the available ones through filter-based methods generally improves model performance, in particular for *k*NN and MPNN methods, with an overall better classification accuracy of the four protein subsets compared to the full protein set. Machine learning algorithms are indeed known to suffer from important decrease of the prediction accuracy when faced with many unnecessary features. Among the different feature subsets, the best classification was obtained with the 29-protein consensus list generated from the three individual ones, with a correct classification rate ranging from 84.2% (3/19 samples incorrectly classified) with *k*NN and RBFNN methods, to 100% with SVM. The three misclassified samples included two CM (AP2 and AP3) and one UM (AS13) samples. With MPNN, only AP3 was incorrectly assigned to the UM group, providing a 94.7% correct classification rate.

**Figure 2:**
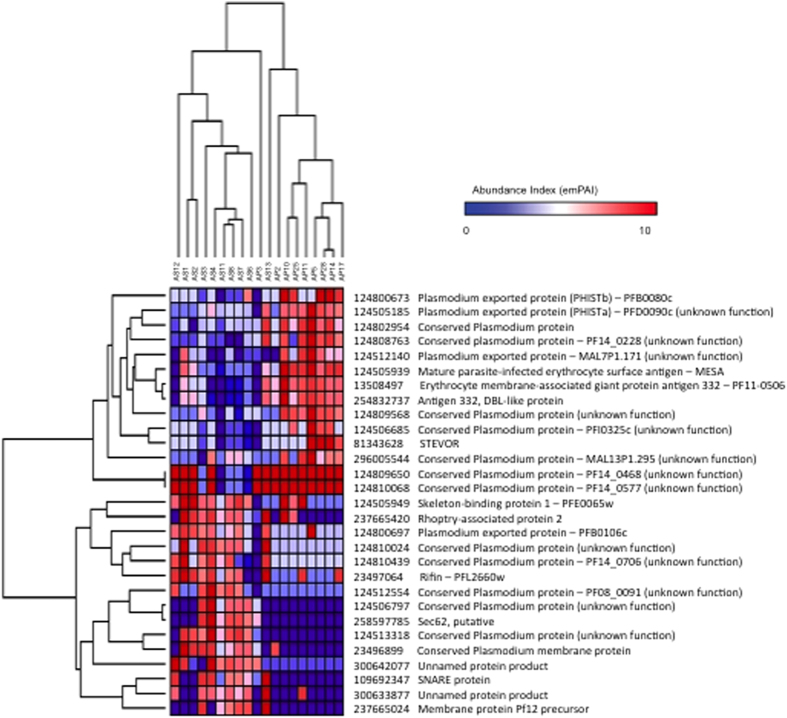
Hierarchical clustering analysis based on the expression profile of the 29 discriminatory proteins in the set of 19 samples. Both samples and proteins were clustered using average linkage clustering, and with Pearson correlation as similarity metric. The samples are shown horizontally (columns), the proteins vertically (rows). The dendrograms represent the distances between clusters. In the heat map of protein expression patterns, expression levels are represented in the color scale of blue (low expression) to red (high expression).

**Figure 3:**
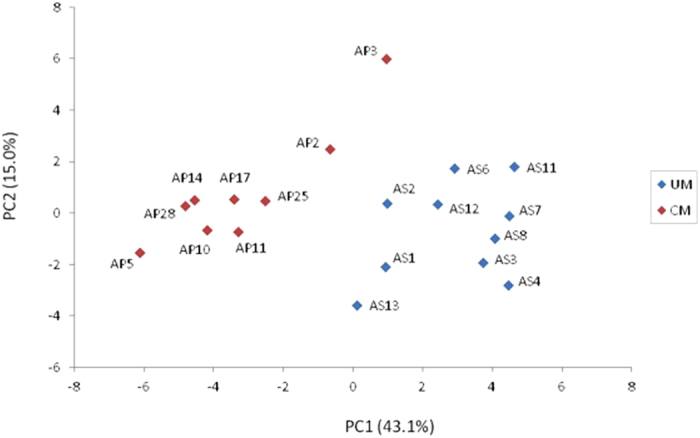
Principal component analysis based on the expression profiles of the 29 discriminatory proteins in the set of 19 samples. Blue and red dots represent uncomplicated malaria (UM) and complicated malaria (CM) samples, respectively. Each axis represents a principal component (PC1 and PC2) with the percentage of the total variance it explains. The next two components (PC3 and PC4) explained 9.2% and 7.1% of total variance, respectively.

Unsupervised hierarchical clustering of the 19 samples based on the expression profiles of these 29 discriminatory proteins essentially separated samples into two main clusters roughly corresponding to the CM and UM clinical status, except for AP3 and AS13 (Fig. 2). The clustering analysis partitioned proteins into two main groups of 14 and 15 proteins over- and under-expressed in CM samples, respectively, as compared to UM (Fig. 2). Similarity in protein expression profiles among the 19 samples was summarized in a scatterplot of the two first principal components of the PCA (Fig. 3). The first principal component (PC1), explaining the largest variation (43.1%), clearly differentiated CM and UM samples.

## Discussion

We aimed to identify membrane proteins specifically expressed or over-expressed in CM. To this end, we used a mass-spectrometry-based proteomic approach to compare membrane and hypothetical proteins of *P. falciparum* parasite isolates from two distinct clinical forms of malaria (CM *vs.* UM). Analysis by LC-MS/MS of nine CM and ten UM samples allowed to identify a total of 1097 *Plasmodium* proteins, 499 being membrane or hypothetical proteins.

The overwhelming number of features (proteins) compared to the relatively low number of samples, leading to the so-called “curse of dimensionality”, hampers the ability to identify proteins that best discriminate between the two classes of parasites. Only a few of the *Plasmodium* proteins expressed at the iE membrane are indeed expected to be differentially expressed between CM and UM samples, and to be associated with malaria severity^20^. Efficient feature selection techniques have been developed to handle such an issue, and are increasingly used in the microarray and mass spectrometry fields^21^,^22^. Instead of choosing one particular feature selection method, and accepting its outcome as the final subset, we used three different methods based on univariate (Fisher’s ANOVA and runs test) or multivariate (ReliefF) filtering. Several authors have stressed the importance of not simply use the pure filter approach for feature selection, and to consider the classification algorithm in the selection procedure^21^,^23^. The success of each subset of features as a classifier was evaluated through supervised classification analysis using four different tools, after which the best set was kept. This set included 29 differentially expressed proteins, discriminating CM and UM samples with high accuracy. Unsupervised clustering analysis based on the expression profiles of these 29 proteins clearly distinguished samples according to their clinical status, except for AP3 and AS13. Our analyses were based on the frequency of identification of each protein in our samples. The multiplicities of variants of *Pf*EMP-1 were computed as different proteins, impairing their regrouping within a clinical group^24^,^25^.

Among these 29 proteins, Fifteen were under-expressed in CM, including 10 proteins having unknown function. One of these proteins (124512554-PF08-0091) has also been identified as down regulated from CM isolates in the work of Almelli *and al*.^15^. Four others are proteins involved in the membrane structure (124505949-skeleton-binding protein 1; 237665420-rhoptry-associated protein 2; 109692347-SNARE protein and 237665024-membrane protein Pf12 precursor). Finally, rifin protein (23497064) has been found under-expressed in CM isolates.

Among the 14 proteins over-expressed in CM, we identified the 124505939-MESA/*Pf*EMP2 protein and the erythrocyte membrane-associated giant protein antigen 332 (254832737 and 13508497). The MESA protein has been shown, from transcriptomic and proteomic data, to be over-expressed in CM isolates as compared to PAM isolates^14^. As suggested by the presence of a PEXEL motif, MESA is expressed at the erythrocyte membrane^26^, located at the lower layer of this membrane, and without antigenic function. MESA is likely involved in protein trafficking, and in the export to variant surface antigens (VSA)^27^. Regarding antigen 332, Nilsson *and al.* proposed a model where this antigen is associated to Maurer’s cleft membrane, and complexes with actin. This model suggests that antigen 332 is also involved in protein trafficking, and in VSAs export^28^. Antigen 332 presents a DBL domain^29^ that is recognized by the sera from malaria hyper-immune individuals, suggesting that this domain could be exported at the membrane of the iE, and could be exposed to the immune system^29^,^30^. However, these and many of the other candidates are involved in iE membrane interactions, and the association may be through their role in the rigidity of the iE, which is known to be associated with pathology. Another possibility is that these proteins reflect the level of expression of VSAs on the iE surface, which provides the link to disease.

Two proteins (124505185-PFD0090c and 124800673-PFB0080c) belonging to the PHIST family, and one protein described as exported to the membrane of iE (124512140-MAL7P1.171) also belong to the cluster of proteins over-expressed in CM. The gene encoding MAL7P1.171 was also reported as up-regulated in CM in comparison to asymptomatic malaria samples (fold change value 4.46) by Almelli *and al*.^15^. Similarly, the genes encoding the proteins MAL13P1.295 and PF14_0577 have been shown to be up regulated in CM samples^15^.

Interestingly, the network study revealed that six of the 14 proteins (124800673-PFB0080c; 124505185-PFD0090c; 124512140-MAL7P1.171; 124505939-MESA; 13508497-erythrocyte membrane-associated giant protein antigen 332; 124506685-PFI0325c) identified as associated with CM, interact directly or indirectly with each other or are co-expressed together (Figures S2 and S3).

At the center of this network, we find the PFB0100c-knob-associated histidine-rich protein (KAHRP) and the PFB0090c-RESA-like protein, demonstrating that the proteins identified are likely to be localized at the membrane of infected erythrocytes. The network indicates that the KAHRP protein interacts with a panel of proteins associated to the CM. This also suggests that KAHRP may interact with a given subset of proteins, depending of the clinical presentation of malaria. Nevertheless, these hypotheses must be confirmed by immunology and biochemistry experiments, to assess the recognition of these proteins by the plasma of children living in malaria endemic areas. The proteomics data presented here demonstrate the association of selected proteins in the pathophysiology of CM, providing new insights for the definition of potential new targets for a vaccine against CM.

## Experimental Procedures

### Ethic statement

Ethical clearance was obtained from the Institutional Ethics Committee of the faculty of health science at the Abomey-Calavi University in Benin. Before inclusion, written informed consent was obtained from all adults and children’ guardians. The medical team of each health facility managed patients with adequate anti-malarial treatment according to the national malaria program policy. The methods were carried out in accordance with the relevant guidelines and regulations. Lastly, all experimental protocols were approved by the Institutional Ethics Committee of the faculty of health science at the Abomey-Calavi University in Benin.

### Samples collection

CM and UM samples used in this study were collected at Cotonou in Benin as described^31^. Briefly, CM patients were recruited with a Blantyre score ≤ 2 at the university medical center and the Lagune Mother and Child hospital. UM patients were enrolled at the health center of Come, located 70 km from Cotonou, on the basis of the presence of an axillary temperature ≥ 37.8 °C without severity sign.

All blood samples were matured *in vitro* for 18 to 32 hours. Maturation was checked after 18 h and thereafter every 4 hours to have at least 50% of the parasites were at the late trophozoite and early schizont stages. Samples that did not achieve such a maturation were discarded. In addition, mature parasites were further enriched in the samples by passage through a Macs column, allowing to reach a mean 80% ± 20% mature stages and finally lysed according to Fried *and al*.^17^. The lysate of each sample was transferred in RIPA (Radio-ImmunoPrecipitation Assay) buffer with 2% SDS and 1X protease inhibitor (Roche), and stored at −80 °C. One hundred μg of proteins were reduced, alkylated, and digested with 1 μg/μl of trypsin overnight at 37 °C according to Bertin *and al*.^24^,^31^.

### LC–MS/MS analysis

Analyses were performed using an Ultimate 3000 Rapid Separation liquid chromatographic system (Dionex, The Netherlands) coupled to a hybrid Linear Trap Quadrupole-ORBITRAP Velos mass spectrometer (Thermo Fisher Scientific, San José CA). Peptides were separated on a C18 RP analytical column (3 μm particle size, 100 Å pore size, 75 μm i.d., 50 cm length) with a 240-minute gradient from 99% A (ACN 5%, formic acid 0.1% and H_2_O 95%) to 40% B (ACN 80%, formic acid 0.085% and H_2_O 20%). The LTQ-ORBITRAP mass spectrometer acquired data throughout the elution process and operated in a data dependent scheme as follows: full MS scans were acquired with the ORBITRAP, followed by up to 10 LTQ MS/MS CID spectra on most abundant precursors detected in the MS scan, as described in details previously^24^,^31^.

### Protein identification and compilation of search results

All LC-MS/MS results were analyzed according to Bertin *et al.*^31^. Proteome Discoverer 1.3.0 software (ThermoFisher Scientific, CA, USA) was used in combination with Mascot 2.2.06 and Sequest search algorithms. The precursor mass tolerance was set to 2 ppm and the fragment mass tolerance to 0.45 Da. The probability of false positive was lower than 5%. The search parameters were set as follows: trypsin specificity with 1 missed tryptic cleavage, 2 ppm mass tolerance (redundant), partial oxidation of methionines, complete carbamidomethylation of cysteines. All proteins identified with both database search algorithms, presenting at least two peptides with a score of identification of proteins ≥ 20 for Mascot and an Xcorr ≥ 1.5 for Sequest, were considered as positive hits. As our samples are highly complex and include both human and parasitic proteins, one cannot work only on a database exclusively dedicated to *Plasmodium*, such as PlasmoDB. The high number of masses analyzed in our samples is disproportionate to the limited size of this database, and this is likely to increase the number of false positives. We used an home made database mixing the Human and *Plasmodium falciparum* sequences from NCBI, completed by the *var* genes sequences from the “vardom” database (*http:* // www.cbs.dtu.dk/*services*/*VarDom*/), we then completed by “reverse” sequences. Our databases may not efficiently recognize VSA proteins due to their variant nature, although the invariant regions may be picked up. Care needs to be taken as some VSA families are over-represented in the database, and will originate significant ‘hits’ without being biologically significant.

### Estimating absolute protein abundance values

The protein abundance values were determined according to^31^. The exponentially modified Protein Abundance Index (emPAI)^19^ was calculated by the following formula:


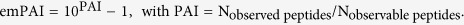


The number of «observable» peptides per protein was calculated by the Protein Digestion Simulator program. To facilitate visualization and comparison, samples with missing emPAI values for a particular protein were assigned half the minimum emPAI value for that protein in the data set^32^. Normalization between samples was performed according to the median of each sample. EmPAI values were then log2 transformed.

### Statistical analysis

Using the set of 19 samples (9 CM and 10 UM), three filter-based methods were applied to select the most relevant features (proteins) that best discriminate between CM and UM samples: Fisher discriminant criterion, Runs test and ReliefF. Fisher filtering follows univariate Fisher’s ANOVA ranking which ranks the input features according to their relevance^33^. Fisher’s criterion takes the mean and the within-class scatter of the groups into account to compare the correlation between features and the class label. Runs filtering is a non-parametric test for predictive feature evaluation^34^. It performs an univariate attribute ranking from the runs test, also known as the Wald-Wolfowitz test, that checks a randomness hypothesis for a two-valued data sequence^35^. In both Fisher and Runs filtering, a significance cut-off *P*-value of <0.05 was used to select the most relevant features. ReliefF is an extension of the popular Relief algorithm^36^. A key idea of ReliefF is to draw samples at random, compute their *k* nearest neighbors of the same class and the opposite class, and adjust a feature weighting vector to give more weight to features that discriminate the sample from neighbors of the opposite class. ReliefF estimates are better than usual statistical feature estimates, like correlation or covariance, because it takes into account features interrelationships. We selected as relevant features those proteins whose weights were higher than the mean of positive weights. The subsets of features selected by each of the three filter-based methods, or by a combination of these, were then presented as input to different supervised classification algorithms to evaluate their performance, and the feature subset yielding the most accurate class assignments (CM *vs.* UM) was kept as the final set.

Supervised class prediction analysis was performed using four machine-learning algorithms: *k*-nearest neighbors^37^, multilayer perceptron neural network^38^, radial basis function neural network^39^, and support vector machine^40^ methods. Our *k*-nearest neighbors classifier was run with *k* = 3 and used a distance-weighted voting scheme with the heterogeneous euclidean overlap metric distance. The neural architecture parameters of the multilayer perceptron were one hidden layer with 20 neurons, and the learning rate was fixed to 0.15. For the support vector regression model with a radial basis function, we used the off-line learning of kernels implemented in Tanagra^41^ with default parameters. Finally, we used a linear support vector machine which implements Platt’s sequential minimal optimization algorithm^42^, and trained the vector support classifier using a polynomial kernel of degree 2. Classification accuracy of the different protein sets was evaluated by leave-one-out cross-validation (LOOCV). The LOOCV estimate of classification accuracy is the overall number of correctly classified samples, divided by the total number of samples.

Unsupervised hierarchical clustering of samples and proteins was performed using average linkage clustering and with Pearson correlation as similarity metric^43^. This clustering technique organizes all data elements into a single tree (referred to as dendrogram) with the highest levels of the tree representing the discovered classes.

As a way to better visualize the data after class prediction analysis, a principal component analysis (PCA)^44^ was applied to the protein expression data and the top components were used to illustrate the similarity in protein expression profiles among CM and UM samples.

All statistical analyses were performed using GenePattern v3.5^45^ and Tanagra v1.4^34^ softwares.

Finally, the network study has been realized with the STRING 10 database allowing the prediction of interaction protein (http://string-db.org). Proteins associated to the CM group were given as input to build the network. The prediction of protein-protein interactions was performed using a high confidence score of 0.70, this score being the probability that a predicted link exists between the proteins, and a limit of no more than 10 interactions.

## Additional Information

**How to cite this article**: Bertin, G. I. *et al.* Proteomic analysis of *Plasmodium falciparum* parasites from patients with cerebral and uncomplicated malaria. *Sci. Rep.* **6**, 26773; doi: 10.1038/srep26773 (2016).

## Supplementary Material

Supplementary Information

## Figures and Tables

**Table 1:** Results of supervised classification analysis on the different sets of proteins before and after filtering.

	Classification accuracy (%) before filtering	Classification accuracy (%) after filtering			
		Fisher’s ANOVA	Runs	ReliefF	29-protein set
No. of features	499	81	6	37	29
Classification algorithm					
*k-nearest neighbors*	63.2%	68.4%	73.7%	68.4%	84.2%
*Multilayer perceptron neural network*	52.6%	94.7%	68.4%	79.0%	94.7%
*Radial basis function neural network*	79.0%	68.4%	63.2%	79.0%	84.2%
*Support vector machine*	79.0%	73.7%	84.2%	100%	100%

Classification accuracy was estimated as the overall number of correctly classified samples divided by the total number of samples through a leave-one-out cross-validation procedure. The highest classification accuracy achieved by each of the five classification algorithms is shown in bold.
