# Supplementary material for: Proteomic analysis of Plasmodium falciparum parasites from patients with cerebral and uncomplicated malaria
Source: Sci Rep. 2016 Jun 1;6:26773. doi: 10.1038/srep26773 (PMC4887788; doi:10.1038/srep26773)

Legend supplementary data

Proteomic analysis of *Plasmodium falciparum* parasites from patients with cerebral and uncomplicated malaria.

Gwladys I. Bertin [1, 2 *], Audrey Sabbagh [1, 2], Nicolas Argy [1, 2, 3, 4], Virginie Salnot [2, 5], Sem Ezinmegnon [6], Gino Agbota [1, 2, 6], Yélé Ladipo [7], Jules M. Alao [7], Gratien Sagbo [8], François Guillonneau [2, 5], Philippe Deloron [1, 2]

[1] Institut de Recherche pour le Développement (IRD), UMR216 - MERIT, Paris, France.

[2] COMUE Sorbonne Paris Cité, Faculté de Pharmacie de Paris, Paris Descartes University, Paris 75006, France.

[3] Parasitology laboratory, Bichat-Claude Bernard hospital, Paris 75018, France.

[4] French national reference center of malaria laboratory, Bichat-Claude Bernard hospital, Paris 75018, France.

[5] 3P5 Proteomics facility, Université Paris Descartes, Paris, France.

[6] Centre d'Étude et de Recherche sur le Paludisme Associé à la Grossesse et l'Enfance (CERPAGE), Cotonou, Bénin.

[7] Paediatric Department, Mother and child hospital (HOMEL), Cotonou, Benin

[8] Paediatric Department, Centre National Hospitalo-Universitaire (CNHU), Cotonou, Benin

Figure S1: Proteins selected by each of the three filter-based methods. Features were ranked according to increasing *p*-value (Fisher's ANOVA and runs test) or decreasing weight (ReliefF). Features selected by a single filter are shown in red, while those selected by two or three filters are shown in orange and yellow, respectively.

Figure S2: STRING 10.0 generated network of CM-associated proteins. The map shows the evidence view of the network with a high confidence (0.7). Colored lines represent the types of evidence for the association (Green: neighborhood; Red: gene fusion; Blue: co-

occurrence; Grey: co-expression; Pink: experiments; Light blue: database; Yellow: text mining; and Purple: homology). The proteins are identified by their gene names near each sphere.

Figure S3: STRING 10.0 generated co-expression of CM-associated proteins. The co-expression view shows the genes that are co-expressed in the same direction. The association score is indicated by a square with a color scale varying from white (no or low co-expression) to brown (high co-expression).

Figure S1

Fisher's ANOVA	
Number of selected features	81
**Feature**	**p-value**
124513318_conserved Plasmodium protein, unknown function	0.0002
124809568_conserved Plasmodium protein, unknown function	0.0003
124810024_conserved Plasmodium protein, unknown function	0.0004
109692347_SNARE protein	0.0012
13508497_erythrocyte membrane-associated giant protein antigen 332	0.0019
124512554_conserved Plasmodium protein, unknown function	0.0025
254832737_Antigen 332, DBL-like protein	0.0062
124808763_conserved Plasmodium protein, unknown function	0.0062
124802954_conserved Plasmodium protein	0.0066
300633877_unnamed protein product	0.0073
23496899_conserved Plasmodium membrane protein	0.0078
124505939_Mature parasite-infected erythrocyte surface antigen (MESA) or PfEMP2	0.0083
258597612_Plasmodium exported protein (PHISTb), unknown function	0.0089
300642077_unnamed protein product	0.0097
237665024_membrane protein Pf12 precursor	0.0108
23496290_conserved Plasmodium protein	0.0113
124513434_conserved Plasmodium protein, unknown function	0.0121
258597772_conserved Plasmodium protein, unknown function	0.0122
124800697_Plasmodium exported protein	0.0123
300536441_unnamed protein product	0.0126
124505801_conserved Plasmodium protein, unknown function	0.0126
160669_S-antigen precursor	0.0128
124802369_conserved Plasmodium protein	0.0129
237665420_rhoptry-associated protein 2	0.0130
258596969_conserved Plasmodium protein	0.0130
81343628_STEVOR	0.0132
124506797_conserved Plasmodium protein, unknown function	0.0132
124809472_conserved Plasmodium protein, unknown function	0.0135
124513290_conserved Plasmodium protein, unknown function	0.0136
258597785_Sec62, putative	0.0153
23496170_conserved Plasmodium protein	0.0166
124511686_conserved Plasmodium membrane protein, unknown function	0.0170
74911696_RING finger protein PFF0165c	0.0172
124506685_conserved Plasmodium protein, unknown function	0.0177
23496149_PfGCN20	0.0178
124809650_conserved Plasmodium protein, unknown function	0.0181
124810068_conserved Plasmodium protein, unknown function	0.0182

Runs test	
Number of selected features	6
**Feature**	**p-value**
124800697_Plasmodium exported protein	0.0014
124505187_Plasmodium exported protein (PHISTb), unknown function	0.0059
124505185_Plasmodium exported protein (PHISTa), unknown function	0.0059
124512554_conserved Plasmodium protein, unknown function	0.0198
124810024_conserved Plasmodium protein, unknown function	0.0198
124505939_Mature parasite-infected erythrocyte surface antigen (MESA) or PfEMP2	0.0198

ReliefF	
Number of selected features	37
**Feature**	**Weight**
124513318_conserved Plasmodium protein, unknown function	0.357129
109692347_SNARE protein	0.2586
237665420_rhoptry-associated protein 2	0.2080
300633877_unnamed protein product	0.1911
124809568_conserved Plasmodium protein, unknown function	0.1903
23496899_conserved Plasmodium membrane protein	0.1834
124810024_conserved Plasmodium protein, unknown function	0.1810
300642077_unnamed protein product	0.1745
124800697_Plasmodium exported protein	0.1617
124808763_conserved Plasmodium protein, unknown function	0.1570
124505939_Mature parasite-infected erythrocyte surface antigen (MESA) or PfEMP2	0.1486
124512554_conserved Plasmodium protein, unknown function	0.1448
13508497_erythrocyte membrane-associated giant protein antigen 332	0.1415
124506797_conserved Plasmodium protein, unknown function	0.1222
124505949_skeleton-binding protein 1	0.1134
237665024_membrane protein Pf12 precursor	0.1128
23497064_rifin	0.1104
258597785_Sec62, putative	0.1102
124810439_conserved Plasmodium protein, unknown function	0.1102
81343628_STEVOR	0.1058
1899003_membrane-associated calcum-binding protein	0.1052
124800673_Plasmodium exported protein (PHISTb)	0.1022
254832737_Antigen 332, DBL-like protein	0.1010
124802954_conserved Plasmodium protein	0.0995
124802160_conserved Plasmodium protein	0.0969
124513440_conserved Plasmodium protein, unknown function	0.0966
124505185_Plasmodium exported protein (PHISTa), unknown function	0.0918
86170403_conserved Plasmodium protein, unknown function	0.0796
124513956_conserved protein, unknown function	0.0794
296005544_conserved Plasmodium protein, unknown function	0.0791
124507285_rifin	0.0758
124512140_Plasmodium exported protein, unknown function	0.0753
237665346_Pf13_0192-like hypothetical protein	0.0749
86170346_rifin	0.0710
124810068_conserved Plasmodium protein, unknown function	0.0709
124809650_conserved Plasmodium protein, unknown function	0.0706
124506685_conserved Plasmodium protein, unknown function	0.0700

296005544_conserved Plasmodium protein, unknown function	0.0185
296004822_conserved Plasmodium protein, unknown function	0.0190
124800673_Plasmodium exported protein (PHISTb)	0.0204
86170439_conserved Plasmodium protein, unknown function	0.0216
23496948_conserved Plasmodium protein	0.0217
23496217_conserved Plasmodium protein	0.0224
86171075_conserved Plasmodium protein, unknown function	0.0224
124808483_conserved Plasmodium protein, unknown function	0.0228
23496317_conserved Plasmodium protein	0.0234
23496157_conserved Plasmodium protein	0.0234
296004702_conserved Plasmodium protein, unknown function	0.0238
23496602_conserved Plasmodium protein	0.0239
259495129_Protein PFF0380w	0.0245
23496365_conserved Plasmodium protein	0.0247
264675902_Uncharacterized protein PF14_0444	0.0248
124808362_conserved Plasmodium protein, unknown function	0.0253
300666210_unnamed protein product	0.0272
296005351_conserved Plasmodium protein, unknown function	0.0280
124512140_Plasmodium exported protein, unknown function	0.0280
311816438_unnamed protein product	0.0284
124512544_conserved Plasmodium protein, unknown function	0.0294
124810593_stevor, putative	0.0298
23495975_NOT family protein, putative	0.0318
23496557_conserved Plasmodium protein	0.0319
124513370_conserved Plasmodium protein, unknown function	0.0325
23496358_conserved Plasmodium protein	0.0340
23496268_conserved Plasmodium protein	0.0345
254832645_conserved Plasmodium protein	0.0348
296005337_conserved Plasmodium protein, unknown function	0.0355
296005560_conserved Plasmodium protein, unknown function	0.0358
258549073_conserved Plasmodium protein	0.0361
124505949_skeleton-binding protein 1	0.0368
124505751_conserved Plasmodium protein, unknown function	0.0368
238630798_rhoptry associated membrane antigen	0.0370
124511764_conserved Plasmodium protein, unknown function	0.0386
23497064_rifin	0.0398
124505181_Plasmodium exported protein (PHISTb), unknown function	0.0399
238630760_rhoptry associated membrane antigen	0.0403
124810439_conserved Plasmodium protein, unknown function	0.0439
124507099_conserved Plasmodium protein, unknown function	0.0450
258597113_conserved Plasmodium protein	0.0457
124513746_conserved Plasmodium protein, unknown function	0.0470
124512900_conserved Plasmodium protein, unknown function	0.0485
124505785_conserved Plasmodium protein, unknown function	0.0493

Figure S2

Network with a high confidence (0.7)

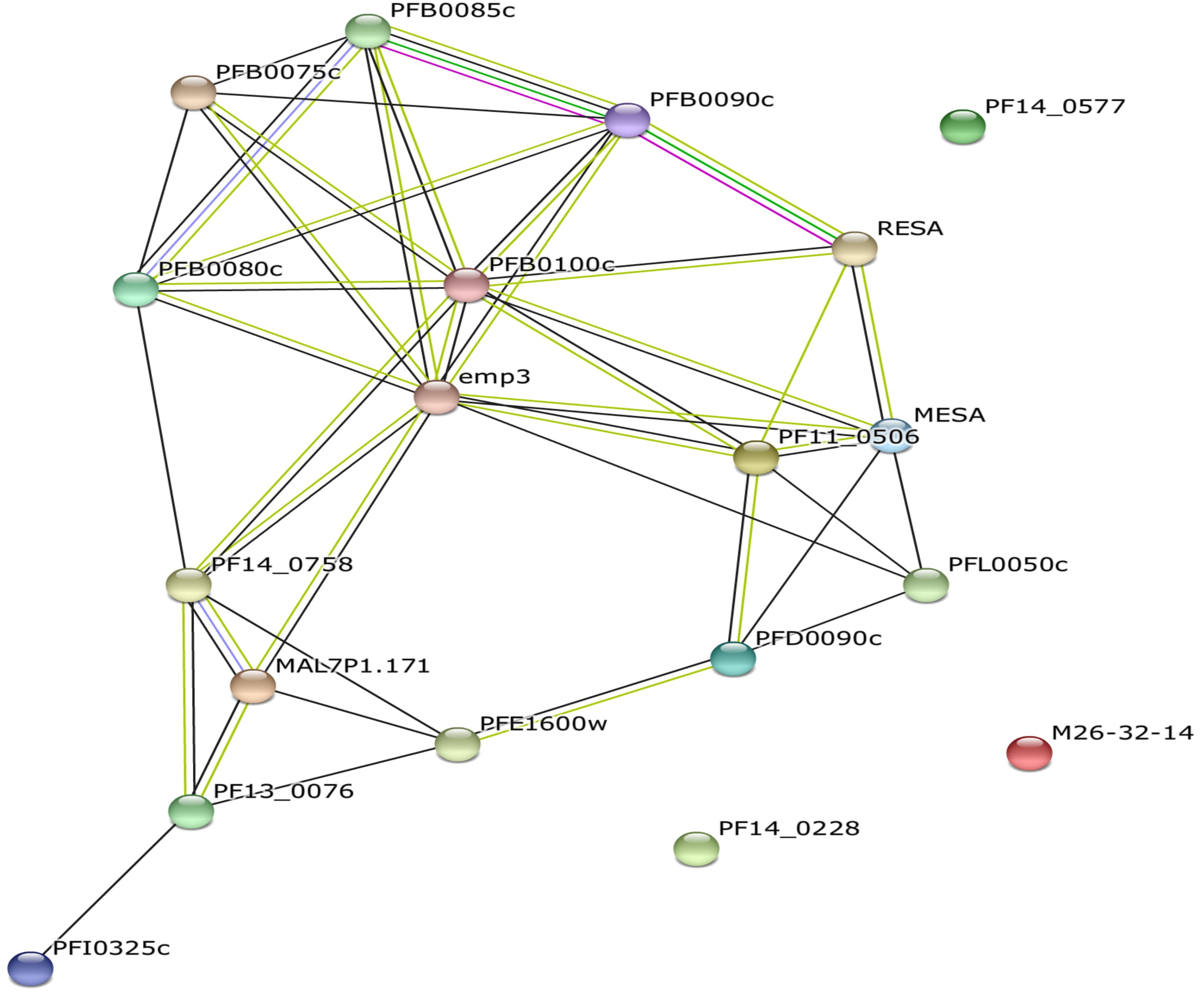

Figure S3

Co-expression

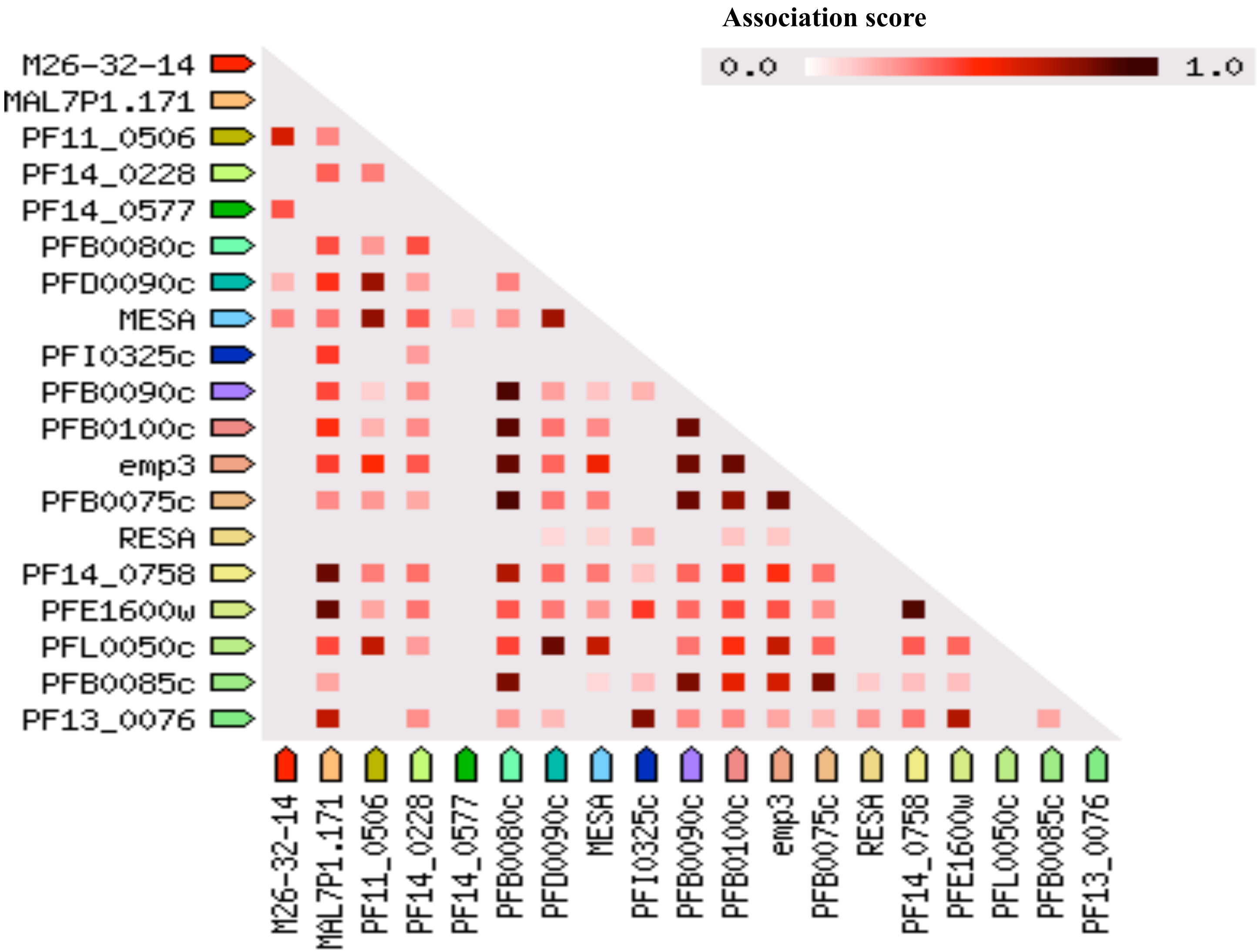

Supplement: Supplementary Information [file srep26773-s1.pdf]
